# M2 macrophages promote myofibroblast differentiation of LR-MSCs and are associated with pulmonary fibrogenesis

**DOI:** 10.1186/s12964-018-0300-8

**Published:** 2018-11-23

**Authors:** Jiwei Hou, Jingyan Shi, Ling Chen, Zhongyang Lv, Xiang Chen, Honghui Cao, Zou Xiang, Xiaodong Han

**Affiliations:** 1 Immunology and Reproduction Biology Laboratory & State Key Laboratory of Analytical Chemistry for Life Science, Medical School, Nanjing University, Hankou Road 22, Nanjing, 210093 China; 2 Jiangsu Key Laboratory of Molecular Medicine, Nanjing University, Nanjing, 210093 China; 3 Department of Health Technology and Informatics, Faculty of Health and Social Sciences, The Hong Kong Polytechnic University, Hung Hom, Kowloon, Hong Kong, China

**Keywords:** Idiopathic pulmonary fibrosis (IPF), M2 macrophages, Lung resident mesenchymal stem cells (LR-MSCs), Myofibroblast differentiation

## Abstract

**Background:**

Idiopathic pulmonary fibrosis (IPF) is a devastating disease characterized by the histopathological pattern of usual interstitial pneumonia and is associated with a high mortality rate. Recently, lung resident mesenchymal stem cells (LR-MSCs) have been identified as an important contributor to myofibroblast activation in pulmonary fibrosis. Macrophages are also believed to play a critical role in pulmonary fibrosis. However, the underlying connections between LR-MSCs and macrophages in the pathogenesis of pulmonary fibrosis are still elusive.

**Methods:**

In this study, we investigated the interaction between LR-MSCs and macrophages using a bleomycin-induced mouse pulmonary fibrosis model and a coculture system.

**Results:**

Here, we show that blocking pulmonary macrophage infiltration attenuated bleomycin-induced pulmonary fibrosis. In addition, as determined by flow cytometry, we discovered that the recruited macrophages in fibrotic lungs of bleomycin-treated mice were mainly M2 macrophages. In particular, we found that M2, rather than M1 macrophages, promoted myofibroblast differentiation of LR-MSCs. Moreover, we demonstrated that suppression of the Wnt/β-catenin signaling pathway could attenuate myofibroblast differentiation of LR-MSCs induced by M2 macrophages and bleomycin-induced pulmonary fibrosis. Tissue samples from IPF patients confirmed the infiltration of M2 macrophages and activation of Wnt/β-catenin signaling pathway.

**Conclusion:**

In summary, this study furthered our understanding of the pulmonary fibrosis pathogenesis and highlighted M2 macrophages as a critical target for treating pulmonary fibrosis.

**Electronic supplementary material:**

The online version of this article (10.1186/s12964-018-0300-8) contains supplementary material, which is available to authorized users.

## Background

Idiopathic pulmonary fibrosis (IPF) is one of the most common forms of interstitial lung diseases characterized by the deposition of interstitial collagen and other extracellular matrix, leading to dyspnea, cough, impaired lung function, and death [1–3]. IPF has a poor prognosis, with a median survival of approximately 3 years from the time of diagnosis [4], and the prevalence of IPF rises dramatically with age. However, therapeutic options to alter the course of this disease remain lacking. As the etiology of IPF is unknown, it is highly desirable to unravel the mechanisms underlying the pathogenesis of pulmonary fibrosis, which may facilitate the development of novel clinical strategies.

In pulmonary fibrosis, the accumulation of fibroblasts and α-SMA^+^ myofibroblasts is largely responsible for the production of collagen within alveolar structures [5]. Therefore, defining the cellular origin of these cells is critical for understanding the pathobiology of pulmonary fibrosis. Recent evidence suggests that lung resident mesenchymal stem cells (LR-MSCs) are precursors of myofibroblasts and also can be induced to differentiate into many other cell types that may participate in lung repair or contribute to the development of pulmonary diseases [6, 7]. Therefore, the roles and fate of LR-MSCs during development in pathological conditions in the lung have attracted substantial attention [8–10]. In particular, the differentiation of LR-MSCs is sensitive to the microenvironment to which these cells are exposed [11].

Inflammatory responses are associated with the development of IPF and inflammatory cells, especially macrophages, that play an important role in the regulation of microenvironment after lung injury [12]. Macrophages are present in virtually all the tissues of the body and play crucial roles in both acute and chronic pulmonary pathologies including cytotoxicity and fibrosis [13–15]. Macrophages are highly plastic and assume their functional phenotype dependent on the inflammatory signals they encounter in the tissue microenvironment [16, 17]. Macrophages can be classified as M1 or M2 subtypes depending on their functional phenotypes [18], although there is a continuum of macrophage polarization beyond the simplified, discrete, in vitro-based classification system. The classically activated macrophages (i.e. M1 macrophages) induced by lipopolysaccharide (LPS), are characterized by production of mediators aimed at eliminating foreign materials and debris [19, 20]. The alternatively activated macrophages (M2 macrophages) which are induced by IL-4 are known to release mediators that down regulate the inflammatory response and promote the resolution of injury and tissue repair [21, 22]. Importantly, M1/M2 macrophage polarization has been associated with the progression of fibrotic diseases [23, 24]. However, the underlying connections between LR-MSCs and macrophage polarization in the pathogenesis of pulmonary fibrosis are still elusive.

The Wnt/β-catenin signaling is an evolutionarily conserved signal pathway that has been demonstrated to play a crucial role in cell fate decision of mesenchymal stem cells in fibrotic disease [25, 26]. This pathway includes a group of ligands that act as intercellular signaling molecules, and importantly, the induction of Wnt ligands by macrophages has been reported to be critical for stem cell regeneration following damage [27, 28]. Nevertheless, the possible involvement of the macrophage phenotype in the activation of Wnt signaling pathways in LR-MSCs has not been investigated.

In the current study, we first investigated the effect of recruited macrophages on the development of bleomycin-induced pulmonary fibrosis. Next, we evaluated the kinetics of in vivo macrophage polarization in the pathogenesis of pulmonary fibrosis. We also examined the effects of macrophages with various activation phenotypes on the differentiation of LR-MSCs. Importantly, the roles of Wnt/β-catenin signaling pathways in myofibroblast differentiation of LR-MSCs and progression of pulmonary fibrosis were examined.

## Materials and methods

### Chemicals

Bleomycin was purchased from Nippon Kayaku (Tokyo, Japan). Clodronate and control liposome were purchased from clodronateliposomes.org (Vrije University, Netherlands). Enzyme-linked immunosorbent assay (ELISA) kits for Wnt7a was purchased from Cusabio (no. CSB-EL026141MO, Wuhan, China). Salinomycin (a specific Wnt/FZD/LRP5 complex inhibitor) was purchased from Medchemexpress (no. HY-15597, Monmouth Junction, NJ). Antibodies used in this study were listed in Additional file 1: Table S1.

### Animals and treatment

Male C57BL/6 (9–10 weeks old) mice were purchased from the Medical School of Yangzhou University (Yangzhou, China). All mice were maintained under standard conditions with free access to water and laboratory rodent food. Bleomycin-induced mouse pulmonary fibrosis model was established as described by us previously [29]. Fifty microliters of clodronate or control liposome was injected intratracheally every three days starting two days before the injection of bleomycin, until the end of the experiment. Mice were sacrificed 21 days after bleomycin instillation.

To evaluate the effect of Wnt/β-catenin signaling on bleomycin-induced pulmonary fibrosis, we injected mice with salinomycin, a small-molecule Compound that specifically inhibits Wnt/β-catenin, as previously described [30]. Specifically, 7 days after administration with bleomycin, mice were intraperitoneally administered with 5 mg/kg salinomycin or vehicle three times a week. In this study, mice were randomly divided into four groups (*n* = 12). Group 1 received saline treatment; group 2 received 5 mg/kg salinomycin; group 3 received 5 mg/kg bleomycin and vehicle; and mice in group 4 received 5 mg/kg bleomycin and 5 mg/kg salinomycin. Mice were sacrificed 21 days after bleomycin instillation. Mouse lungs were obtained for the analysis of immunofluorescence analyses, q-PCR and western blotting.

All procedures carried out on animals were approved by the Animal Care and Use Committee of Nanjing University under the animal protocol number SYXK (Su) 2009–0017.

### Cell line culture and macrophage polarization

RAW 264.7 macrophage-like cells, obtained from the cell bank of Chinese Academy Sciences (Shanghai, China), were cultured as described previously [31]. RAW 264.7 cells in exponential growth were plated at a density of 1 × 10^5^ cells/ml. After overnight incubation, cells were treated with 10 ng/ml LPS (Sigma–Aldrich no. L2880, St. Louis, MO) or 10 ng/ml IL-4 (Peprotech no. AF-214-14, Rocky Hill, NJ) for 24 h to induce M1 and M2 macrophage differentiation, respectively [32–34].

### Indirect coculture system

Isolation of LR-MSCs was performed as previously reported [35]. Indirect coculture system was established using cell culture inserts (0.4 mm PET, 4.5 cm^2^, Millipore). LR-MSCs and RAW macrophages were each plated at 1 × 10^5^ cells/ml. LR-MSCs were plated in the lower chamber and RAW macrophages were plated in the upper chamber. After 72 h coculture, inserts were removed and LR-MSCs were harvested for western blotting and immunofluorescence analyses. Furthermore, for assessing the regulation of Wnt/β-catenin signaling, salinomycin was added to the medium of the cocultured LR-MSCs at a concentration of 1 μM in DMSO, at the beginning of coculture. DMSO was added in the control culure.

### RNA interference

Commercially available siRNA for the mouse Wnt7a (sc-41,115) gene and nonspecific control siRNA (sc-37,007) were purchased from Santa Cruz Biotechnology. Transient transfection was carried out as detailed previously [36].

### Flow cytometric analysis

To determine the macrophage subtypes in fibrotic lungs, lower right lungs were minced with fine scissors, and enzymatically digested with 0.1% type I collagenase (Sigma no. C0130) for 1 h at 37 °C with gentle agitation every 20 min. The single cell suspension was refiltered through a 40-μm nylon mesh after digestion to remove connective tissue, after which the cells were washed with D-Hanks and collected by centrifugation by 300×g for 5 min. Cells were incubated with PE-conjugated F4/80 antibodies or APC-conjugated CD206. Flow cytometry was performed on a FACS CaliburTM flow cytometer and the data were analyzed using Paint-A-Gate software (Becton Dickinson).

In addition, flow cytometric analyses were used to determine the macrophage polarization in vitro. RAW 264.7 cells after stimulation were incubated with fluorescent antibodies at 37 °C for 40 min in the dark followed by two washes with PBS. The antibodies used were: PE-conjugated anti-CD68, PE-conjugated anti-CCR7, and APC-conjugated anti-CD206.

### Human lung tissue

Surgical biopsy specimens from IPF lung tissue samples were obtained from Nanjing Drum Tower Hospital. Patient controls were selected to be similar in age to IPF patients with nonfibrotic lung disorders. Lung biopsy samples were processed by standard techniques for western blotting, Immunohistochemistry, and immunofluorescence analyses. All protocols concerning the use of patient samples in this study were approved by the Ethics Committee of Nanjing Drum Tower Hospital.

### Immunohistochemistry, hematoxylin-eosin (H&E) and Masson’s trichrome stain

Immunohistochemistry was performed on 4-μm, paraffin-embedded lung tissue and mounted on polylysine-coated slides. The slides were cleared of paraffin and subjected to antigen retrieval (10.2 mM sodium citrate, 0.05% Tween 20, pH 6.0, 10 min). Next, quenching of endogenous peroxidase activity was achieved by incubation with 3% (*v*/v) H_2_O_2_ for 10 min, followed by incubation with rat anti-Sca-1, rat anti-F4/80, rabbit anti-iNOS, or rabbit anti-CD206 at 4 °C overnight. The DAB Substrate System (DAKO) was used to reveal the immunohistochemical staining. In addition, the slides were stained with H&E for structured observation, or with Masson’s trichrome stain for detection of collagen deposits according to the instructions by the manufacturer (KeyGen no. KGA224/KGMST-8003, Nanjing, China).

### Immunofluorescence

Immunofluorescence analyses of LR-MSCs or lung tissues were performed as described previously [37]**.** The following primary antibodies were employed: rat anti-Sca-1, mouse anti-α-smooth muscle actin (α-SMA), rabbit anti-collagen I, mouse anti-CD206, rabbit anti-F4/80, and rabbit anti-Wnt7a. Alexa Fluor 488-conjugated goat anti-mouse antibody, Alexa Fluor 488-conjugated goat anti-rat antibody, Alexa Fluor 594-conjugated goat anti-mouse antibody and Alexa Fluor 594-conjugated goat anti-rabbit antibody (Invitrogen no.A-11001, A-11006, A-11032, and A-11037, respectively, Carlsbad, CA, 1:200 dilution) was used as a secondary antibody. Nuclei were stained with 4′,6-diamidino-2-phenylindole (DAPI) (Sigma no. D9542). The images were observed under confocal fluorescence microscope (Olympus, Tokyo, Japan) with the Z-stack technique (0.8 μm/layer).

### Quantitative real-time polymerase chain reaction (Q-PCR)

The RNA extraction was performed as previously described [38]. The sequences of primer pairs used in this assay are shown in Additional file 1: Table S2. Q-PCR was conducted by amplifying 20 μl of diluted cDNA with the SYBR Green Q-PCR kit (Vazyme no.Q711–02, Nanjing, China) on an ABI ViiA 7 Q-PCR System (Applied Biosystems, Waltham, MA). Each sample was run in triplicate and PCR reactions without the addition of the template were used as blank controls. The relative quantification of the expression of the target genes was measured using glyseraldehyde-3-phosphate dehydrogenase (GAPDH) mRNA as an internal control.

### Western blotting, coimmunoprecipitation (co-IP) and ELISA analyses

Proteins were purified from either LR-MSCs or lung tissues. Western blotting analyses of cellular lysates were performed as previously described [39]. Cytosolic and nuclear extracts were prepared with nuclear and cytosolic extraction reagent kit (Invent Biotechnologies no.SC-003, Eden Prairie, MN) according to the manufacturer’s instructions. Proteins were separated using 12% SDS-polyacrylamide gel electrophoresis and were electrophoretically transferred to polyvinylidene fluoride (PVDF) membranes using standard procedures. Next, these plots were incubated overnight at 4 °C with rabbit anti-collagen I, rabbit anti-β-catenin, mouse anti-α-SMA, rabbit anti-Wnt7a, mouse anti-Frizzled-1, rabbit anti-iNOS, mouse anti-CD206, rabbit anti-Histone H3, rabbit anti-Sca-1, and rabbit anti-GAPDH. Horseradish peroxidase-conjugated goat anti-rabbit/mouse IgG (Boster no. BA1056/BA1050, Wuhan, China, 1:10,000 dilution) was used as a secondary antibody. Co-IP was performed by using 800 μg of protein from lysates of LR-MSCs. Anti- Frizzled-1 antibody was used as the precipitating antibody to isolate Frizzled-1 from lysates, followed by western blotting to identify whether Wnt7a can bind to Frizzled-1 on the membrane using anti-Wnt7a antibody. Blots were re-probed with anti-Frizzled-1antibody to confirm equal protein loading. Co-IP with mouse IgG served as a negative control. Wnt7a levels in cell supernatant were measured by ELISA kits according to the manufacturer’s instructions.

### Statistical analyses

SPSS 18.0 (SPSS, Chicago, IL) was used for statistical analysis. The data are presented as mean values ± SD. The Student’s t test was used for paired comparisons.

For the comparison of three or more groups, one-way ANOVA was used for the comparison, which was followed by Duncan’s post hoc test. A *p* value of less than 0.05 was considered statistically significant.

## Results

### The myofibroblast differentiation of LR-MSCs is closely correlated with pulmonary fibrogenesis

**Fig. 1:**
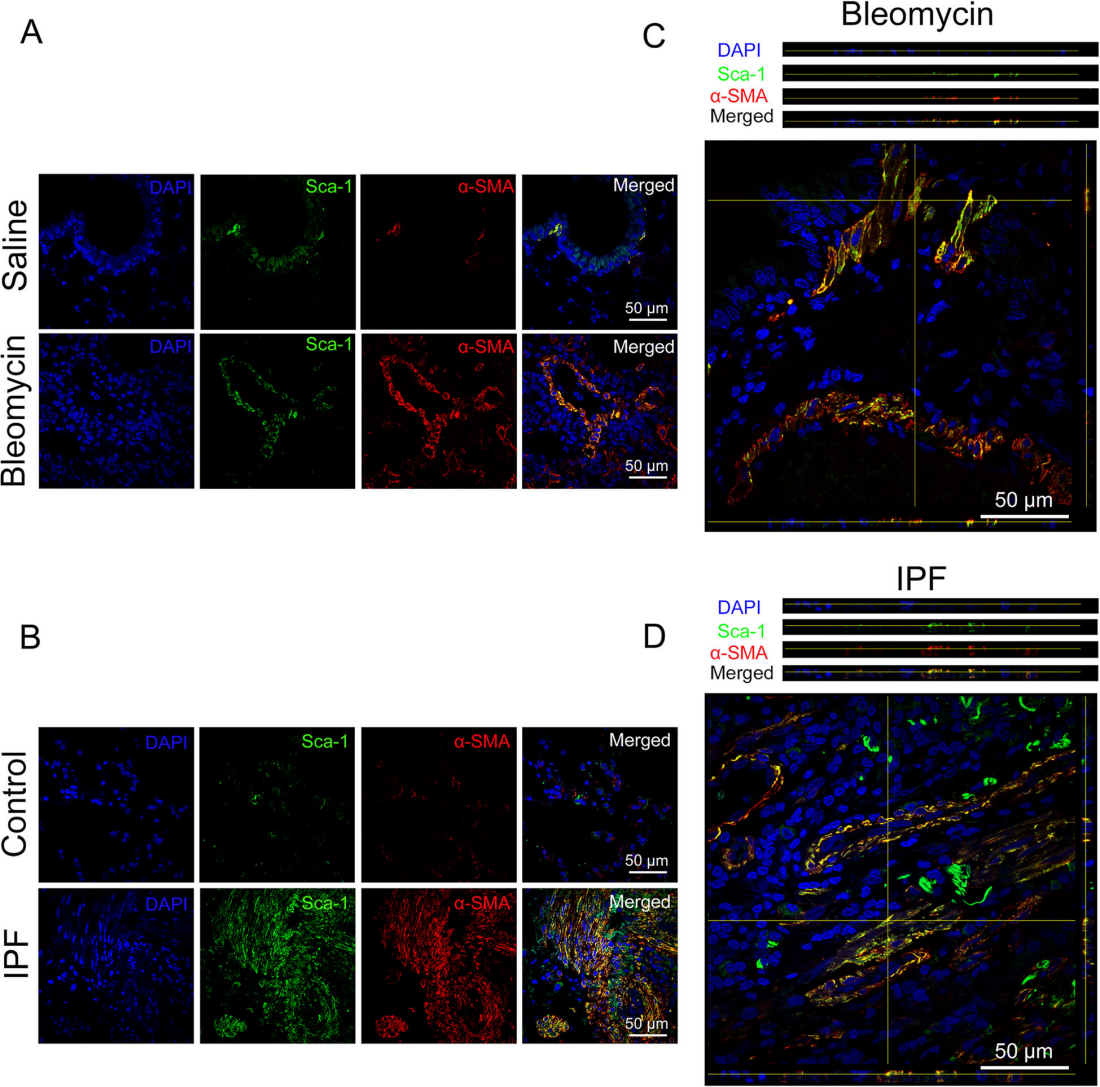
Myofibroblast differentiation of LR-MSCs occurs in pulmonary fibrogenesis. **a** Mice (*n* = 10 in each group) received either saline or bleomycin (5 mg/kg body weight) intratracheally. Mice were sacrificed 21 days later. The colocalization of Sca-1 (mesenchymal stem cell marker) and α-SMA (myofibroblast marker) was determined by immunofluorescence assay. **b** The colocalization of Sca-1 and α-SMA in human normal lung tissues (upper panels, *n* = 7) and IPF lung tissues (lower panels, *n* = 7) was determined by immunofluorescence assay. **c** and **d** Representative z-stack image analysis showed specific overlay of double immunostaining. Sca-1^+^/α-SMA^+^ cells in specific ordinate were analyzed in z-stack with optimal interval range of 0.8 μm

Following intratracheal spray of bleomycin, the expression of α-SMA and collagen I was increased (Additional file 1: Figure S1), suggesting the occurrence of fibrosis. We next investigated the colocalization of mesenchymal stem cells and mesenchymal markers. Colocalization of Sca-1 and myofibroblast marker α-SMA in the endothelium layer of pulmonary arterioles and microcapillary vessels was observed at day 21 after bleomycin injection (Fig. 1a). Specific overlay of Sca-1^+^/α-SMA^+^ cells was examined by z-stack analysis [40] (Fig. 1c). In addition, the above results were also confirmed in lung tissues of IPF patients (Fig. 1b and d). Our findings suggest that myofibroblast differentiation of LR-MSCs may be a source of myofibroblast accumulation in pulmonary fibrogenesis.

### Macrophage infiltration contributes to the development of bleomycin-induced pulmonary fibrosis

**Fig. 2:**
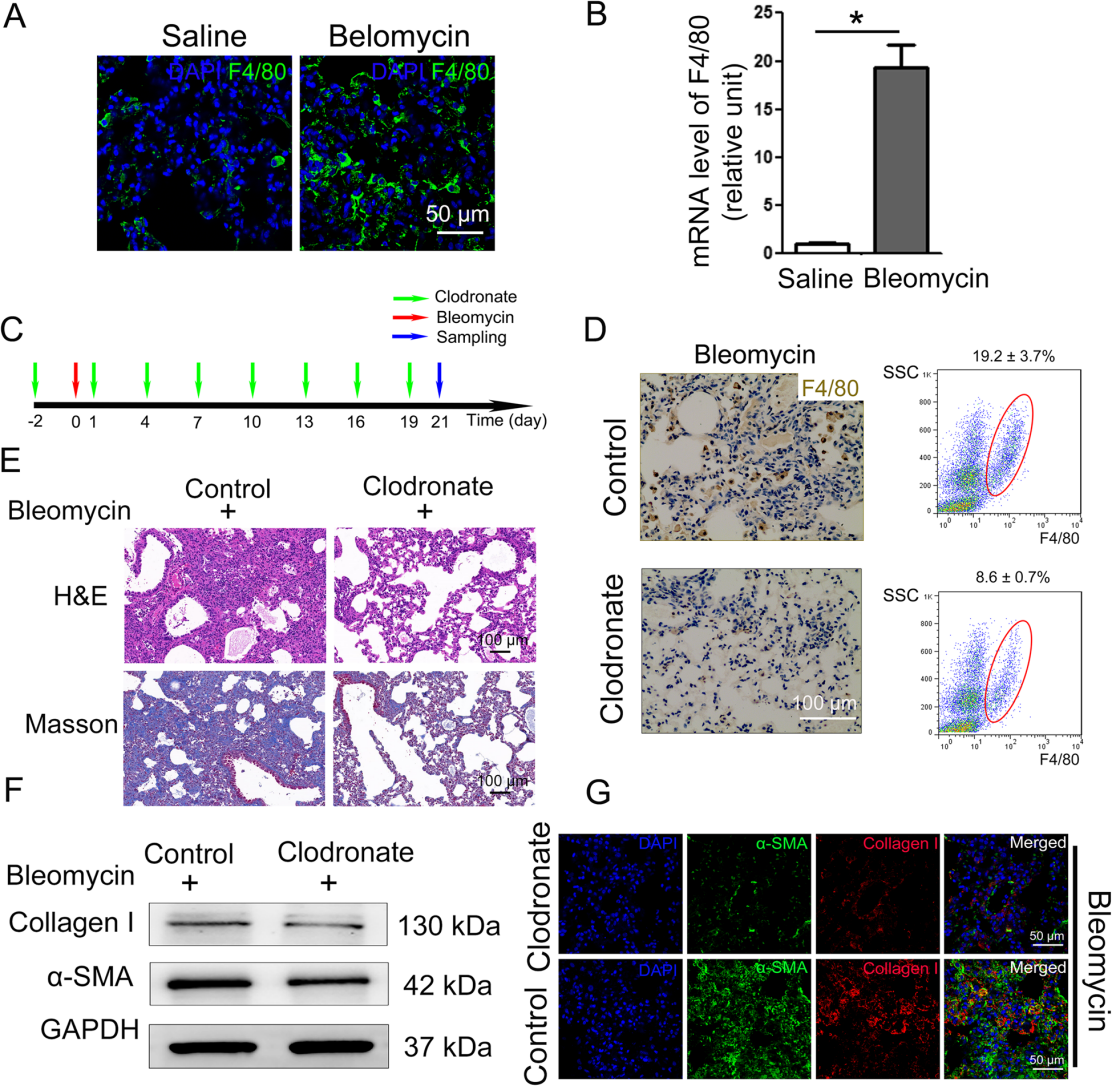
Inhibition of macrophage infiltration inhibits the development of pulmonary fibrosis in bleomycin-treated mice. **a** Mice (*n* = 10 in each group) received either saline or bleomycin (5 mg/kg body weight) intratracheally. Mice were sacrificed 21 days later. The expression of F4/80 in lung tissues was measured by immunofluorescence assay. Representative images are shown. **b** Expression of F4/80 mRNA in lung tissues was measured by q-PCR. Results are expressed as means ± SD (*n* = 5; *p < 0.05 vs. Saline). **c** A myeloid-specific ablating liposome, clodronate, was injected intratracheally every three days starting two days before the injection of bleomycin, and control mice received injections of a control liposome (*n* = 10). **d** The expression of F4/80 in lung tissues was examined by immunohistochemistry (left panels) and the infiltration of macrophages was analyzed by flow cytometric analysis (right panels). **e** Pulmonary fibrosis was determined by hematoxylin-eosin (H&E) staining and collagen I was revealed by Masson’s trichrome staining. Representative micrographs of histology are shown. **f** The protein levels of α-smooth muscle actin (α-SMA) and collagen I in lung tissues was measured by western blotting. **g** The expression of α-SMA and collagen I in lung tissues was measured by immunofluorescence assay

Although inflammatory macrophages have been shown to play an essential role in the progression of fibrosis and tissue remodeling [41, 42], the exact mechanism remains largely unknown. To elucidate the mechanisms by which the recruited macrophages participate in the development of pulmonary fibrosis, we first performed immunostaining for F4/80, a specific marker for macrophages, on lung tissues from saline or bleomycin-treated mice. We found a robust increase in F4/80^+^ cells in mouse lungs following bleomycin treatment (Fig. 2a and b). Thus, these data suggest that the development of bleomycin-induced pulmonary fibrosis is accompanied by robust macrophage infiltration.

To explore whether the recruited macrophages may affect the development of pulmonary fibrosis, we tried to chemically deplete macrophages by clodronate, a myeloid-specific ablating liposome that induces apoptosis of macrophages [43, 44]. Clodronate was injected intratracheally every three days starting two days before the injection of bleomycin (Fig. 2c). Our data demonstrated a marked decline in F4/80^+^ cells in the clodronate-treated mice. We quantified the frequency of F4/80^+^ cells in the dissociated whole lung tissue cells by flow cytometry and found that macrophages decreased from 19.2 ± 3.7% of the total lung cells in the control mice (injected with control liposome) to 8.6 ± 0.7% of the total lung cells in clodronate-treated mice (Fig. 2d). In addition, the mRNA levels of the inflammatory factors (i.e. IL-1β, IL-6 and TNF-α) were decreased in mouse lungs after clodronate treatment (Additional file 1: Figure S2). Taken together, clodronate efficiently reduced the recruitment of inflammatory macrophages in bleomycin-treated mice.

Next, we wanted to determine whether this reduction in inflammatory macrophages may affect the development of bleomycin-induced pulmonary fibrosis. We found that treatment with clodronate attenuated the severity of pulmonary fibrosis confirmed by morphological changes assessed by H&E and Masson’s trichrome staining (Fig. 2e). Moreover, the expression of collagen I and α-SMA, the major markers of pulmonary fibrosis, was profoundly decreased in clodronate-treated mice (Fig. 2f and g). These data suggest that infiltrating macrophages exacerbated bleomycin-induced pulmonary fibrosis.

### Infiltrated macrophages in fibrotic lungs are mainly M2 macrophages

**Fig. 3:**
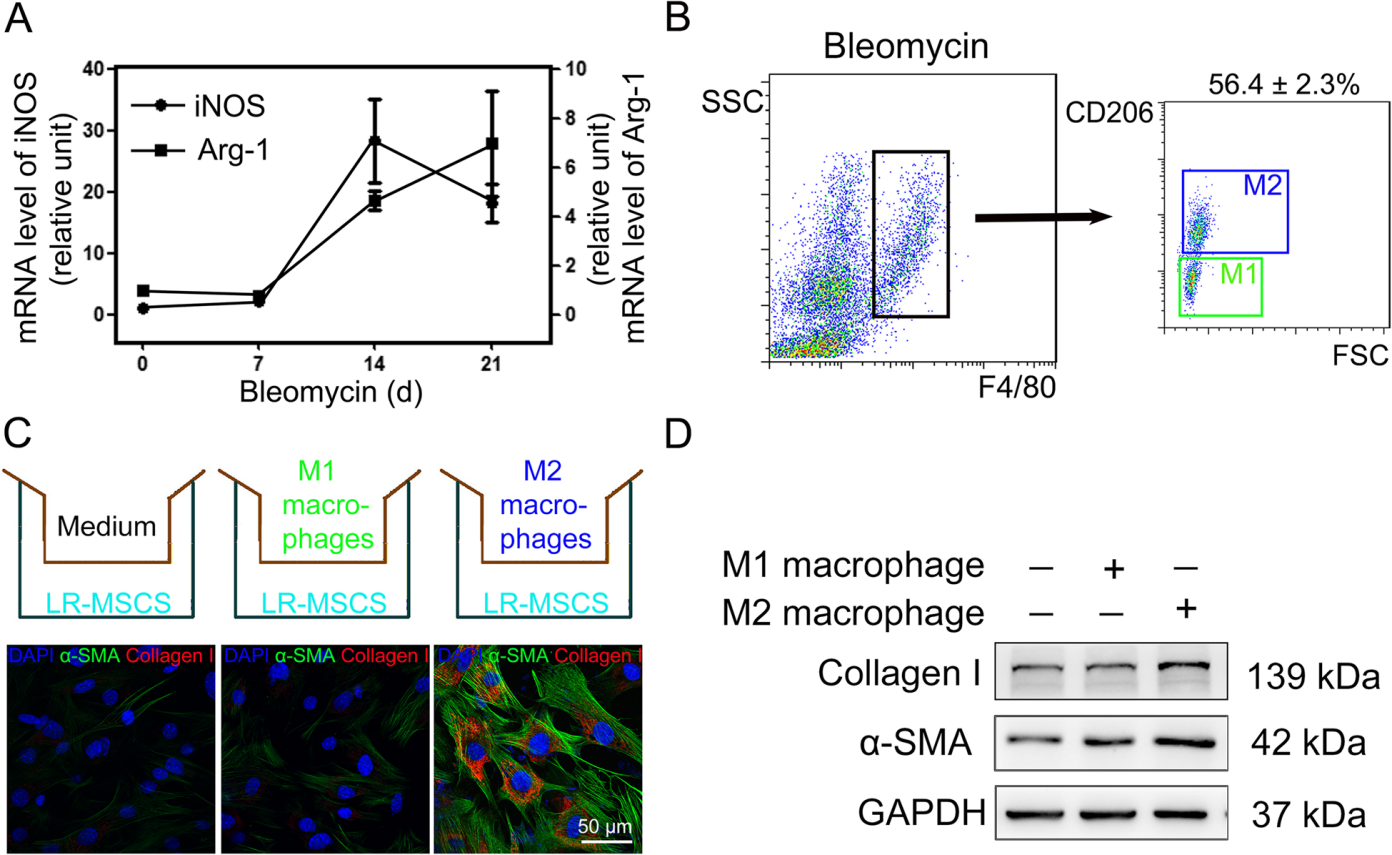
M2 macrophage infiltration predominates in fibrotic lungs of bleomycin-treated mice. Mice (*n* = 10 in each group) received either saline or bleomycin (5 mg/kg body weight) intratracheally. **a** Lung tissues obtained at the indicated time points postinjection were analyzed by q-PCR for mRNA expression of inducible nitric oxide synthase (iNOS) and arginase (Arg-1), marker genes for M1 and M2 polarization, respectively. **b** Representative gating strategy is shown to identify pulmonary macrophage subsets from whole lung-digests in bleomycin-treated mice. M1 and M2 macrophages were analysed by using flow cytometry for the M2 macrophage marker, CD206 in the F4/80^+^ cell fraction (*n* = 3). **c** and **d** Purified primary LR-MSCs were cocultured with M1 macrophages or M2 macrophages polarized from mouse macrophages (RAW264.7) in vitro, or with control medium, in a transwell system. The expression of α-smooth muscle actin (α-SMA) and collagen I in LR-MSCs was measured by immunofluorescence assay **c** and western blotting (**d**)

As macrophages demonstrate two distinct functional phenotypes (M1 and M2 macrophages) that are responsible for various pathophysiological processes, we thus evaluated the kinetics of in vivo macrophage polarization in mice following intratracheal injection of bleomycin. We first examined the mRNA expression of the primary M1 and M2 polarization markers including inducible nitric oxide synthase (iNOS) and arginase (Arg-1) in lung tissues of bleomycin-treated mice. At 14 days postinjection, pulmonary macrophages were strongly M1 polarized, as they expressed high levels of iNOS mRNA and very low levels of Arg-1 mRNA (Fig. 3a). At 21 days postinjection, the overall polarization of macrophages shifted to M2 as Arg-1 expression was increased and iNOS expression was decreased (Fig. 3a). Next, we confirmed our finding using flow cytometric analysis of surface markers that could differentiate M2 and M1 macrophages. Our results demonstrated that the frequency of CD206^+^ M2 macrophages in the F4/80^+^ cell population increased to (56.4 ± 2.3%) macrophages (F4/80^+^) in fibrotic lungs 21 days after treatment (Fig. 3b). These data confirmed the predominance of the recruited macrophages in fibrotic lungs were M2 macrophages.

### M2 macrophages promote myofibroblast differentiation of LR-MSCs through the Wnt/β-catenin signaling pathway

The differences between recruited macrophage subsets suggest that M1 and M2 macrophages may have different effects on the differentiation of LR-MSCs. To test the functional involvement of M2 macrophages in fibrotic pathology, we first induced macrophage polarization in vitro by using LPS or IL-4 into either M1 or M2 phenotypes, respectively (Additional file 1: Figure S3). Nest, we cocultured M1 macrophages (LPS-stimulated, CD68^+^ CCR7^+^CD206^−^) or M2 macrophages (IL-4–stimulated, CD68^+^ CCR7^−^CD206^+^) with purified primary LR-MSCs in a transwell system (Fig. 3c). After 72 h coculture, incubation with M1 macrophages did not modulate the differentiation of LR-MSCs. Interestingly, incubation with M2 macrophages dramatically promoted myofibroblast differentiation of LR-MSCs evidenced by the expression of α-SMA (Fig. 3c and d), suggesting that M2 macrophages, rather than M1 macrophages, are able to promote myofibroblast differentiation of LR-MSCs in the pathogenesis of pulmonary fibrosis.

**Fig. 4:**
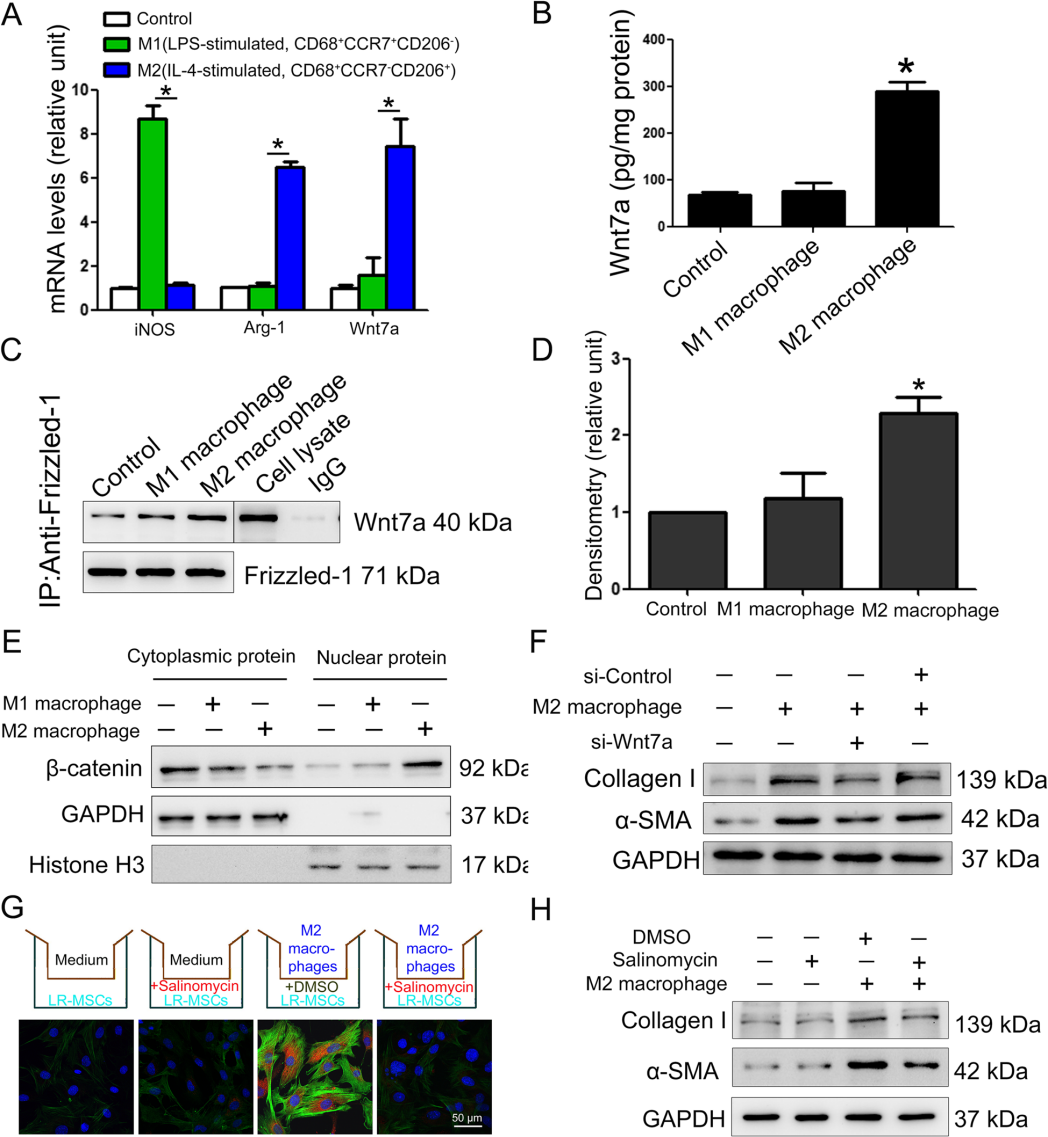
M2 macrophages promote myofibroblast differentiation of LR-MSCs through the Wnt/β-catenin signaling pathway. **a** RAW 264.7 cells were treated with LPS (10 ng/ml) or IL-4 (10 ng/ml) for 24 h to induce M1 and M2 macrophage differentiation, respectively. The mRNA expression levels of inducible nitric oxide synthase (iNOS, M1 macrophage marker), arginase (Arg-1, M2 macrophage marker), and Wnt7a in differentiated macrophage subtypes were determined by q-PCR. Results are expressed as means ± SD (*n* = 5; *p < 0.05 vs. M1 macrophage). **b** Wnt7a levels in the culture supernatant of differentiated macrophage subtypes were determined by ELISA. Data were expressed as means ± SD (*n* = 5; *p < 0.05 vs. control). **c** LR-MSC lysates were subjected to coimmunoprecipitation (Co-IP) with anti-Frizzled-1 antibody, and the blot was probed with anti-Wnt7a antibody. Moreover, blots were re-probed with anti-Frizzled-1 antibody to confirm equal protein loading. Co-IP with mouse IgG served as a negative control. The presence of Wnt7a in the cell lysate was detected by western blotting, serving as a positive control. **d** The ratios of Wnt7a/Frizzled-1 were determined by densitometry and were expressed as means ± SD (*n* = 3; *p < 0.05 vs. control). **e** The nuclear translocation of β-catenin was evaluated by measuring protein levels in the cytosolic and nuclear extracts. Histone H3 and GAPDH were used as loading controls for nuclear and cytoplasmic proteins, respectively, and also used as a control for the purity of the preparation. **f** M2 macrophages were transfected with control or Wnt7a siRNA and then cocultured with LR-MSCs in a transwell system. The expression of α-smooth muscle actin (α-SMA) and collagen I in LR-MSCs was measured by western blotting. **g** and **h** Purified primary LR-MSCs were cocultured with M2 macrophages or control medium in a transwell system. In some of the cocultured LR-MSCs as indicated 1 μM salinomycin (a specific Wnt/FZD/LRP5 complex inhibitor) or the solvent DMSO was added to the medium of the cocultured LR-MSCs to block Wnt/β-catenin signaling. Expression of α-smooth muscle actin (α-SMA) and collagen I on LR-MSCs was measured by immunofluorescence assay (**g**) and western blotting (**h**)

Next, we screened for the candidate factors that may be released from M2 macrophages to affect myofibroblast differentiation of LR-MSCs. The likely candidates were Wnt signaling ligand, which is reported to play important roles both in the differentiation of stem cells and in the development of fibrotic diseases [45, 46]. Among the numerous factors tested (selected genes shown in Additional file 1: Figure S4), we observed a profound increase of Wnt7a mRNA (Fig. 4a). Moreover, Wnt7a protein was also substantially enriched in the cell culture supernatant of M2 macrophages (Fig. 4b). Consistent with these in vitro data, we further observed an increase in the level of Wnt7a protein in fibrotic lungs and at least some of the Wnt7a expression could be localized to M2 macrophages (Additional file 1: Figure S5 and S6). Using Co-IP we showed the interaction of Wnt7a with Frizzled-1 in LR-MSCs (Fig. 4c and d), which may induce the activation of Wnt/β-catenin signaling pathway (Fig. 4e) leading to myofibroblast differentiation of LR-MSCs (Fig. 4f). These data suggest that the canonical Wnt/β-catenin signaling pathway in LR-MSCs may be regulated by M2 macrophages in fibrotic lungs.

The Wnt/β-catenin signaling cascade is initiated when a Wnt molecule binds to the frizzled receptor and to the lipoprotein receptor-related protein 5 or 6 (LRP5/6) coreceptors, forming a ternary complex at the cell surface. Therefore, we next investigated the effect of Wnt/β-catenin signaling on the myofibroblast differentiation of LR-MSCs induced by M2 macrophages using salinomycin which inhibits Wnt/β-catenin signaling by acting on the Wnt/Fzd/LRP complex [30]. As expected, salinomycin inhibited the nuclear translocation of β-catenin (Additional file 1: Figure S7). Notably, the myofibroblast differentiation of LR-MSCs facilitated by M2 macrophages was also profoundly inhibited by salinomycin (Fig. 4g and h).

### Bleomycin-induced pulmonary fibrosis was alleviated by inhibiting the Wnt/β-catenin signaling pathway

**Fig. 5:**
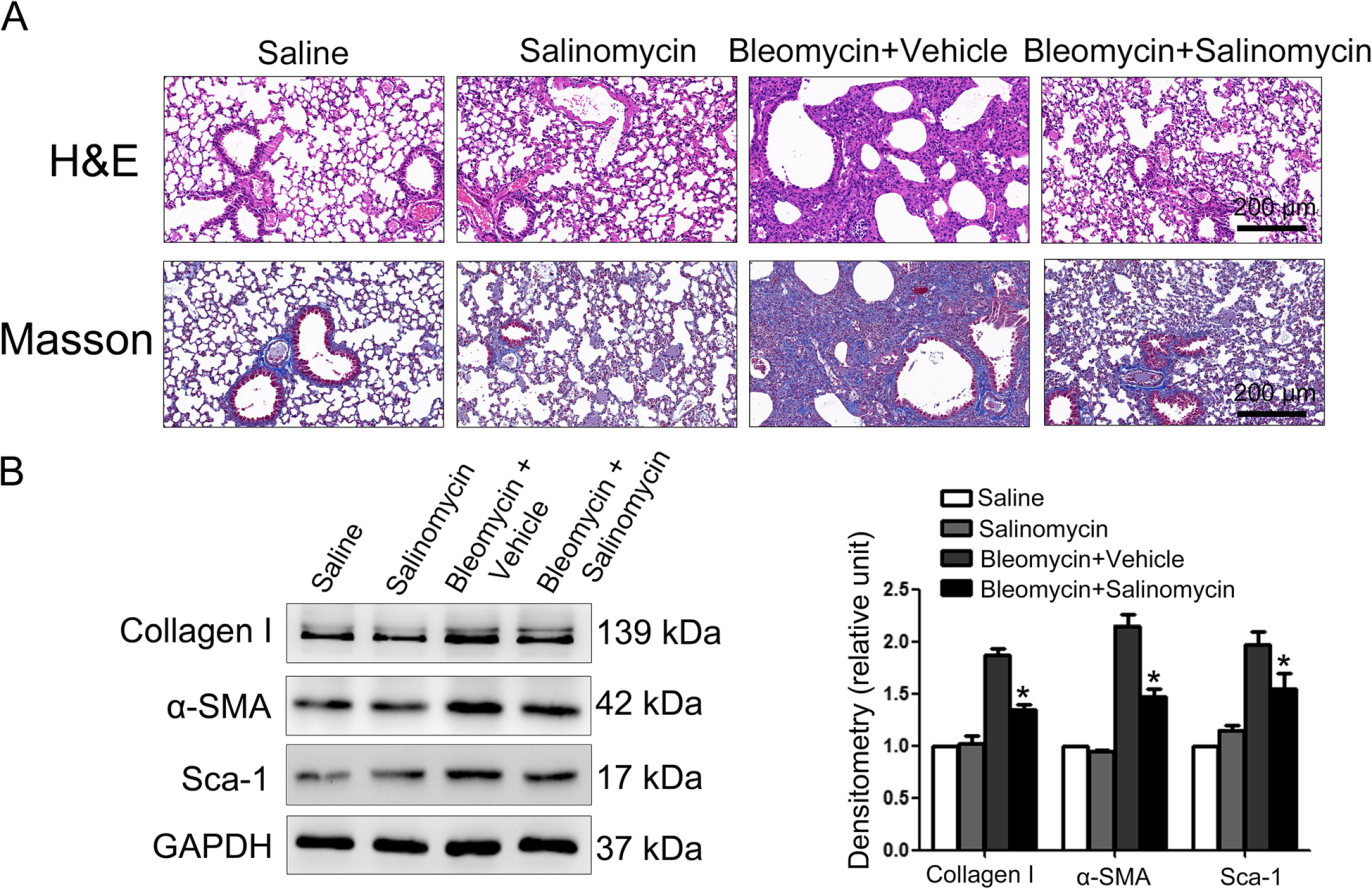
Blockade of the Wnt/β-catenin signaling pathway inhibits the development of bleomycin-induced pulmonary fibrosis. Mice (*n* = 10 in each group) were intraperitoneally injected with vehicle (10% DMSO/saline) or 5 mg/kg salinomycin three times a week as indicated 7 days after the administration of bleomycin. Mice were sacrificed at day 21 after bleomycin instillation. **a** Pulmonary fibrosis was determined by hematoxylin-eosin (H&E) staining and collagen I was revealed by Masson’s trichrome staining. Representative micrographs of histology are shown. **b** The expression of collagen I, α-smooth muscle actin (α-SMA), and Sca-1 in lung tissues was measured by western blotting. The expression levels were quantified with ImageJ (right panels; *n* = 3). GAPDH was used as a loading control. Results are normalized to the expression of each individual protein in the control which is given a value of 1 and are expressed as means ± SD (*p < 0.05 vs. Bleomycin + Vehicle)

We further explored the effect of Wnt/β-catenin signaling on bleomycin-induced pulmonary fibrosis. As shown in Fig. 5a, administration of salinomycin reduced the severity of pulmonary fibrotic lesions and collagen deposition as assessed by H&E and Masson’s trichrome staining. In addition, bleomycin-induced up-regulation of Sca-1, α-SMA, and collagen I was also suppressed by salinomycin (Fig. 5b).

### Increased infiltration of M2 macrophages and activation of Wnt/β-catenin signaling were observed in the lung tissues of IPF patients

**Fig. 6:**
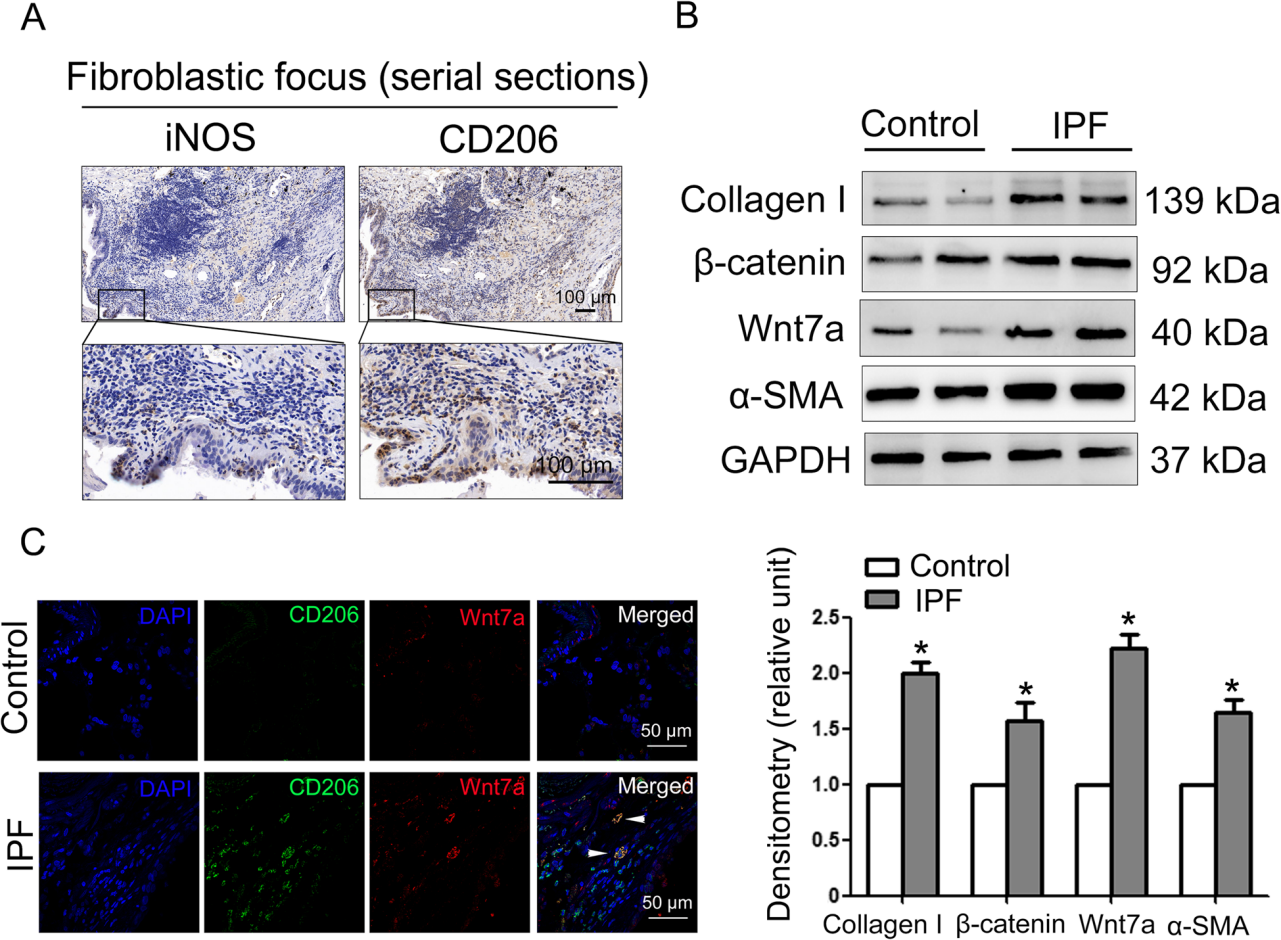
M2 macrophages infiltration predominates in human IPF lung tissues. **a** The expression of iNOS (M1 macrophage marker) and CD206 (M2 macrophage marker) in IPF fibroblastic focus were examined by immunohistochemistry. Representative images are shown (*n* = 7). **b** The expression of collagen I, β-catenin, Wnt7a, and α-smooth muscle actin (α-SMA) in human IPF lung tissues was measured by western blotting. The expression levels were quantified with ImageJ (lower panels; *n* = 3). GAPDH was used as a loading control. Results are normalized to the expression of each individual protein in the control which is given a value of 1 and are expressed as means ± SD (*p < 0.05 vs. Control). **c** Expression of Wnt7a protein in CD206^+^ M2 macrophages was measured by immunofluorescence assay. Arrow indicates individual cells positive for both CD206 (green) and Wnt7a (red). Representative images are shown

To verify our findings in clinical samples, we next extended our mouse experiments to the examination of IPF patients. As shown in Fig. 6a, M2 macrophages in human IPF lung tissues outnumbered M1 macrophages, supporting that M2 macrophages might play an important role in human pulmonary fibrogenesis. Moreover, the expression of β-catenin and Wnt7a was also upregulated, suggesting the activation of Wnt/β-catenin signaling in human patients (Fig. 6b). In addition, the colocalization of Wnt7a and M2 macrophages was observed (Fig. 6c).

**Fig. 7:**
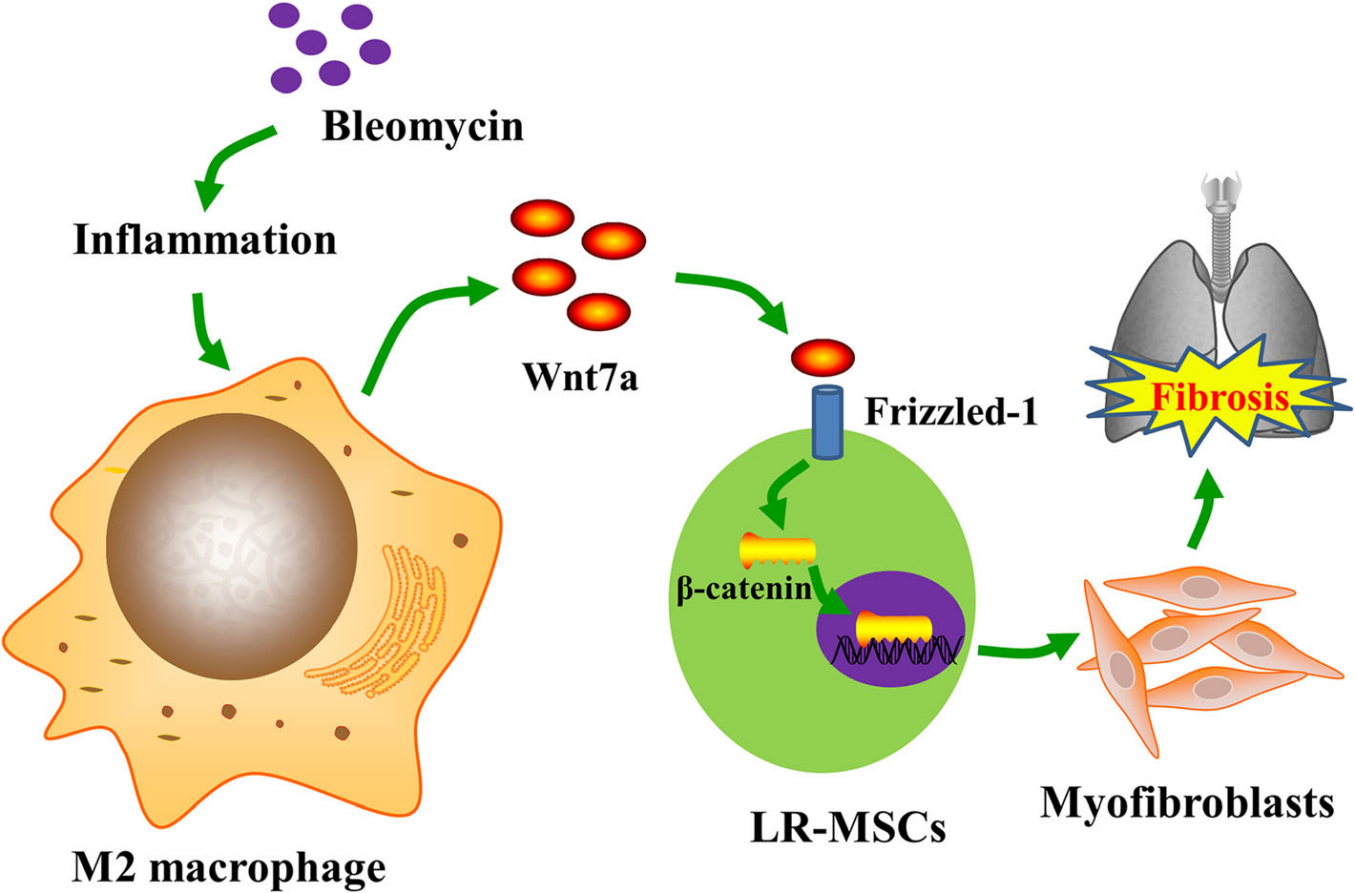
Schematic representation for hypothetical M2 macrophage-induced myofibroblast differentiation of LR-MSCs. Bleomycin induces severe local inflammation. The recruited M2 macrophages release high levels of Wnt7a, which promotes myofibroblast differentiation of LR-MSCs and pulmonary fibrosis through activating the Wnt/β-catenin signaling pathway

Taken together, our data highlight a pivotal role of M2 macrophage in the progression of pulmonary fibrosis, possibly by the activating Wnt/β-catenin signaling pathway to promote myofibroblast differentiation of LR-MSC (Fig. 7).

## Discussion

Idiopathic pulmonary fibrosis (IPF) is a chronic, progressive lung disease that has a death rate worse than that of many cancers. The pathogenesis of IPF, for which there is still no effective clinical therapy, remains unknown. Recent studies have demonstrated that pulmonary macrophage is involved in the pathogenesis of pulmonary fibrosis [41]. In the current study, we found a substantial infiltration of inflammatory macrophages in fibrotic lungs of bleomycin-treated mice. In a loss-of-function experiment using clodronate to deplete macrophages, we showed that macrophage infiltration is necessary for the development of bleomycin-induced pulmonary fibrosis. It is of note that the alveolar walls of the clodronate-treated group appeared mild degree of fracture and some parts of the fractured alveolar fused, although the severity of pulmonary fibrosis was attenuated. The reason for this phenomenon is probably due to the fact that clodronate injected intratracheally every three days is invasive. This problem can be solved in the future by improving the mode of administration.

Macrophages comprise a heterogeneous population: M1 (classically activated) and M2 (alternatively activated). M1 macrophages are typically associated with inflammation which could have a role in the onset or progression of fibrotic disease; whereas M2 macrophages are involved in matrix deposition and tissue remodeling [47]. Given the complicated heterogeneity in polarization and function of macrophages, it is reasonable to suppose that different subsets of macrophages play different roles in pulmonary fibrogenesis. Here, we showed that M1/M2 ratio inversed from day 14 to day 21 after bleomycin injection, suggesting functional adaptation. Moreover, we used a specific M2 macrophage marker CD206 to differentiate M1 and M2 macrophages by flow cytometry. We found that recruited macrophages in fibrotic lungs are mainly M2 macrophages. These results suggest that a tissue microenvironment rich in M2 macrophages may play an important role in the development of pulmonary fibrosis.

Recent evidence suggests that adult lung tissue contains a population of LR-MSCs that have multiple differentiation potential, and these cells are increasingly recognized as a major source of fibrosis-associated myofibroblasts in pulmonary fibrosis [9, 10]. The present study observed colocalization of Sca-1 and mesenchymal marker α-smooth muscle actin (α-SMA) in lung tissues after bleomycin injection. Our data demonstrated that the myofibroblast differentiation of LR-MSCs occurs during pulmonary fibrogenesis, suggesting that LR-MSCs could play important roles in the development of pulmonary fibrosis.

As the differentiation of LR-MSCs is sensitive to the microenvironment to which these cells are exposed, we set out to investigate the effect of different macrophage subtypes on the differentiation of LR-MSCs. In our study, LPS and IL-4 were used to induce the polarization of M1 and M2 macrophage, respectively. We found that LPS and IL-4 stimulation differentially induced complete M1 and M2 polarization, defined by surface marker expression. Then we established an in vitro Transwell coculture system consisting of activated macrophages and LR-MSCs, and we found that M2, rather than M1 macrophages, can promote the myofibroblast differentiation of LR-MSCs, evidenced by the expression of α-SMA. No single marker reliably identifies all myofibroblast, however, α-SMA, especially when expressed at a high level, remains a widely recognized marker of myofibroblasts [48]. Although M2 macrophages have been studied for their central role in fibrosis and cell proliferation during tissue repair [42, 49], our study provides strong evidence, which was previously unidentified, that they may also have a promoting effect on myofibroblast differentiation of LR-MSCs.

Next, we investigated the possible signaling pathways in LR-MSCs that may be targeted by macrophage-induced LR-MSCs differentiation. The Wnt/β-catenin signaling has been extensively implicated in the differentiation of mesenchymal stem cells and progression of fibrotic diseases [50, 51]. Recent studies have shown that macrophage-induced activation of Wnt/β-catenin signaling affects the differentiation of hepatic progenitor cells in chronic liver diseases [28]. In this study, we detected a significant increase of Wnt7a protein in fibrotic lungs and specifically in M2 macrophages. M2 macrophages-derived Wnt7a may possibly act on the surrounding LR-MSCs through autocrine or paracrine modes of action. Once Wnt7a binds to the Frizzled-1 of LR-MSCs, the Wnt/β-catenin signaling pathway can be activated triggering stem cell differentiation [45]. Indeed we observed the activation of Wnt/β-catenin signaling in myofibroblast differentiation of LR-MSCs (Fig. 4d). Using Co-IP we showed the interaction of Wnt7a with Frizzled-1 on LR-MSCs, which may induced the nuclear translocation of β-catenin. Moreover, we demonstrated that inhibition of Wnt/β-catenin signaling could suppress the M2 macrophage induced myofibroblast differentiation of LR-MSCs.

Then we investigated the effect of disrupting Wnt/β-catenin signaling on bleomycin-induced pulmonary fibrosis. It is generally considered that the lung response after bleomycin has two successive phases: inflammatory phase (day 0 to day 7) and fibrotic phase (day 14 to day 21). To avoid disrupting bleomycin-mediated inflammation which might also be dependent on Wnt/β-catenin signaling, we injected salinomycin intraperitoneally 7 days after bleomycin administration. The results showed that targeted inhibition of Wnt/β-catenin signaling by salinomycin protects lungs from bleomycin-induced pulmonary fibrosis. This result was consistent with previous reports [51–54], but in contrast with several reports that have shown that a reactivation of Wnt/β-catenin signaling led an attenuation of experimental emphysema [55, 56]. The reason for this discrepancy may result from distinct mechanisms of Wnt/β-catenin signaling in the pathogenesis of these two chronic pulmonary diseases, which has been well discussed previously [57].

In addition, it is important to point out that the antifibrotic efficacy of the Wnt/β-catenin inhibitor salinomycin may be attributed to other possible mechanisms besides inhibiting myofibroblast differentiation of LR-MSCs. First, another proposed source of fibrotic cells is epithelial cells that undergo epithelial-mesenchymal transition (EMT), a process frequently mediated by TGF-β1. Of note, it has been shown that Wnt/β-catenin pathway blocking-mediated attenuation of pulmonary diseases was accompanied by a decreased expression of TGF-β1in vivo. These results suggested that salinomycin may exert its antifibrotic action through reducing the expression of TGF-β1 and further inhibiting EMT. Secondly, the macrophage M1 – M2 polarization balance may be affected by salinomycin. It has been shown that a coordinate action of various transcription factors, signaling molecules, and inflammatory modulators was involved in regulating macrophage polarization. The presently known molecular determinants of M1 – M2 polarization include members of the STAT, KLF, IRF, PPAR, NF-κB, miRNAs, and HIF families [58].

However, the role of Wnt/β-catenin signaling in macrophage polarization still need to be further clarified in future studies.

Furthermore, our analyses of lung tissues from IPF patients suggested that the infiltration of M2 macrophages was increased concomitantly with the activation of Wnt/β-catenin signaling, in agreement with the results from animal model. These results confirmed the clinical significance of our findings and raised the question of the molecular mechanisms that influence the macrophage M1 – M2 polarization balance in pulmonary fibrogenesis.

## Conclusions

To summarize, we discovered that recruited macrophages in fibrotic lungs of bleomycin-treated mice are mainly M2 macrophages. In particular, we found that M2, rather than M1 macrophages, promote myofibroblast differentiation of LR-MSCs through activating Wnt/β-catenin signaling. Thus, our study not only reveals the relationship between macrophages and LR-MSCs in the pathogenesis of pulmonary fibrosis, but also indicates that modulation of macrophage polarization may be a promising treatment for pulmonary fibrosis. Future studies may focus on dissection of the further downstream pathways in LR-MSCs in response to M2 macrophages and definition of the molecular mechanisms that influence macrophage polarization.

## Additional file

Additional file 1: **Table S1.** Specifications of primary antibodies. **Table S2.** Primers used for q-PCR. **Figure S1.** Pulmonary fibrosis is induced in bleomycin-treated mice. **Figure S2.** Deletion of macrophages inhibits the expression of inflammatory factors. **Figure S3.** Cytokine-induced macrophage polarization. **Figure S4.** M2 macrophages express significant levels of Wnt7a. **Figure S5.** Bleomycin treatment upregulates Wnt7a, and activates Wnt/β-catenin signaling in mouse lung tissues. **Figure S6.** M2 macrophages express Wnt7a in fibrotic lungs of bleomycin-treated mice. **Figure S7.** Effects of salinomycin on the nuclear translocation of β-catenin induced by M2 macrophages in LR-MSCs. (PDF 823 kb)

## Abbreviations

Arg-1: Arginase
iNOS: Inducible nitric oxide synthase
IPF: Idiopathic pulmonary fibrosis
LR-MSCs: Lung resident mesenchymal stem cells
PBS: Phosphate buffered saline
Sca-1: Stem cell antigen-1
α-SMA: α-smooth muscle actin
