# Supplementary material for: M2 macrophages promote myofibroblast differentiation of LR-MSCs and are associated with pulmonary fibrogenesis
Source: Cell Commun Signal. 2018 Nov 23;16:89. doi: 10.1186/s12964-018-0300-8 (PMC6260991; doi:10.1186/s12964-018-0300-8)
Supplement: Supplementary file 1 — Table S1. Specifications of primary antibodies. Table S2. Primers used for q-PCR. Figure S1. Pulmonary fibrosis is induced in bleomycin-treated mice. Figure S2. Deletion of macrophages inhibits the expression of inflammatory factors. Figure S3. Cytokine-induced macrophage polarization. Figure S4. M2 macrophages express significant levels of Wnt7a. Figure S5. Bleomycin treatment upregulates Wnt7a, and activates Wnt/β-catenin signaling in mouse lung tissues. Figure S6. M2 macrophages express Wnt7a in fibrotic lungs of bleomycin-treated mice. Figure S7. Effects of salinomycin on the nuclear translocation of β-catenin induced by M2 macrophages in LR-MSCs. (PDF 823 kb) [file 12964_2018_300_MOESM1_ESM.pdf]

M2 macrophages promote myofibroblast differentiation of LR-MSCs and are associated with pulmonary fibrogenesis

Jiwei Hou[1,2], Jingyan Shi[1,2], Ling Chen[1,2], Zhongyang Lv[1,2], Xiang Chen[1,2], Honghui Cao[1,2], Zou Xiang[3], Xiaodong Han[1,2*]

[1] Immunology and Reproduction Biology Laboratory & State Key Laboratory of Analytical Chemistry for Life Science, Medical School, Nanjing University, Nanjing, 210093, China.

[2] Jiangsu Key Laboratory of Molecular Medicine, Nanjing University, Nanjing, 210093, China

[3] Department of Health Technology and Informatics, Faculty of Health and Social Sciences, The Hong Kong Polytechnic University, Hung Hom, Kowloon, Hong Kong, China.

E-mail address:

Jiwei Hou: houjiwei3707@126.com

Jingyan Shi: 18260098653@163.com

Ling Chen: chenlingcathy@163.com

Zhongyang Lv: zhongyanglv@163.com

Xiang Chen: chenxiang910110@163.com

Honghui Cao: caohonghui92@gmail.com

Zou Xiang: xiang.y.zou@polyu.edu.hk

Mailing address: Immunology and Reproductive Biology Laboratory, Medical College of Nanjing University, Hankou Road 22, Nanjing, 210093, China

***Corresponding author:** Xiaodong Han

E-mail address: hanxd@nju.edu.cn

Tel: +86-25-83686497

Fax: +86-25-83686497

Supplementary Table S1. Specifications of primary antibodies

Vendor	Antibody	Catalog no.	Working dilution
Abcam	Rat anti-Sca1	ab51317	IF, 1:100
	Rabbit anti-Sca-1	ab109211	WB, 1:2,000
	Rabbit anti-F4/80	ab100790	IF, 1:100 IHC, 1:100
	Rabbit anti-GAPDH	ab181602	WB, 1:5,000
	Rabbit anti-iNOS	ab15323	WB, 1:2,000 IHC, 1:100
Proteintech	Rabbit anti-beta-catenin	51067-2-AP	WB, 1:2,000
	Mouse anti-CD206	60143-1-AP	WB, 1:2,000 IF, 1:50
	Rabbit anti-Wnt7a	10605-1-AP	WB, 1:2,000 IF, 1:50
	Rabbit anti-Histone H3	17168-1-AP	WB, 1:1,000
Santa cruz	Mouse anti-Frizzled 1	sc-398082	IP, 1:40
Boster	Rabbit anti-Collagen I	BA0325	WB, 1:1,000 IF, 1:50
	Mouse anti-α-SMA	BM0002	WB, 1:1,000 IF, 1:50
BD Biosciences	PE Rat anti-mouse F4/80	565410	Flow Cyt, 1:100
	PE Rat anti-mouse CD68	566386	Flow Cyt, 1:100
eBioscience	PE Rat anti-mouse CCR7	85-12-1971-82	Flow Cyt, 1:100
	APC Rat anti-mouse CD206	85-17-2061-82	Flow Cyt, 1:100

Supplementary Table S2. Primers used for real-time PCR.

Accession Number	Target gene	Primer sequence (5'-3') Formard	Reverse	Size (bp)	Tm (℃)
NM _007482.3	Arg-1	TTTTAGGGTTACGGCCGGTG	CCTCGAGGCTGTCCTTTTGA	146	59.5
NM _001355722.1	F4/80	TGTCTGAAGATTCTCAAAACATGGA	TGGAGCTTCATAGTTGTAAGGCA	125	58.7
NM _001289726.1	GAPDH	CCCTTAAGAGGGATGCTGCC	TACGGCCAAATCCGTTCACA	124	60
NM_008361.4	IL-1β	TGCCACCTTTTGACAGTGATG	TGATGTGCTGCTGCGAGATT	138	59.5
NM_001314054.1	IL-6	TTCCTCTGGTCTTCTGGAGT	TGTGACTCCAGCTTATCTCTTGG	146	59
NM _001313921.1	iNOS	TCTAGTGAAGCAAAGCCCAACA	CTCTCCACTGCCCCAGTTTT	169	60
NM_001278601.1	TNF-α	CCCTCACACTCACAAACCAC	ACAAGGTACAACCCATCGGC	133	59
NM _001256224.2	Wnt5a	CTCCGGCCCAGAAGCC	AGAAAAACGTGGCCAAAGCC	108	59.5
NM _001363757.1	Wnt7a	GGAGCTCAAAGTGGGGAGTC	CCAGGATCTTGCTTCTCCTTGT	138	60
NM _001163633.1	Wnt7b	TAGGAAGGCCAGTGACCAGA	ACAATGCTCTGTAAGATGGCG	143	59
NM _009290.2	Wnt8a	GCCTATCTGACCTACACCGC	GATGTCTCTCTCGTGGCAGC	155	60
NM _011720.3	Wnt8b	TGACCGGTCCAAAGGCTTAC	CAACGGTCCCAAGCAAACTG	100	60
NM _009518.2	Wnt10a	AGATCGCCATCCATGAGTGC	ACTCTCTCGAAAACCTCGGC	122	60

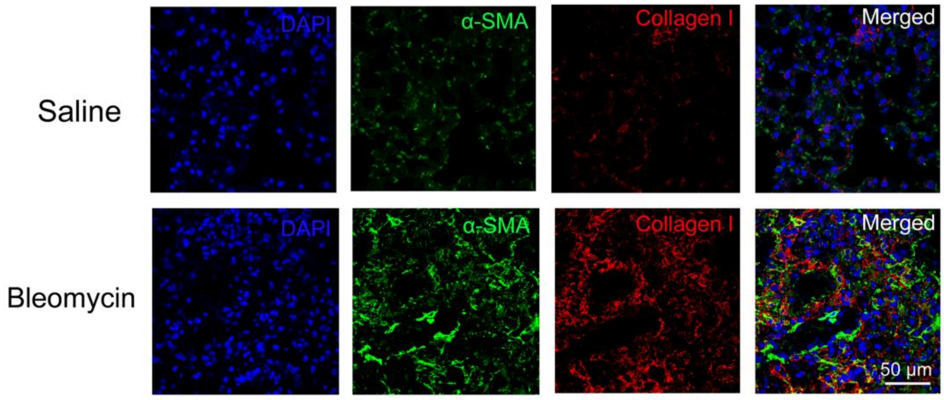

Supplementary Fig.1 Pulmonary fibrosis is induced in bleomycin-treated mice. Mice (n = 10 in each group) received either saline or bleomycin (5 mg/kg body weight) intratracheally. Mice were sacrificed 21 days later. The expression of α-smooth muscle actin (α-SMA) and collagen I in lung tissues was measured by immunofluorescence assay.

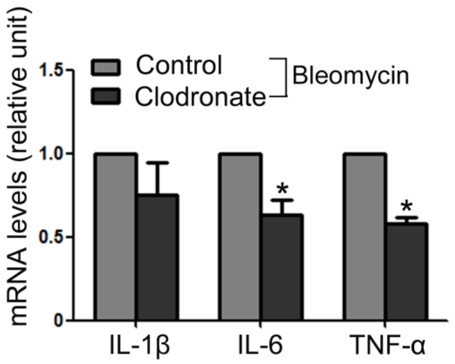

Supplementary Fig.2 Deletion of macrophages inhibits the expression of inflammatory factors. A myeloid-specific ablating liposome, clodronate, was injected intratracheally every three days starting two days before the injection of bleomycin, and control mice received injections of a control liposome (n = 10). Expression of IL-1β, IL-6, and TNF-α in lung tissues were measured by q-PCR. Results are expressed as means ± SD (n = 5; *$p < 0.05$ vs. Control).

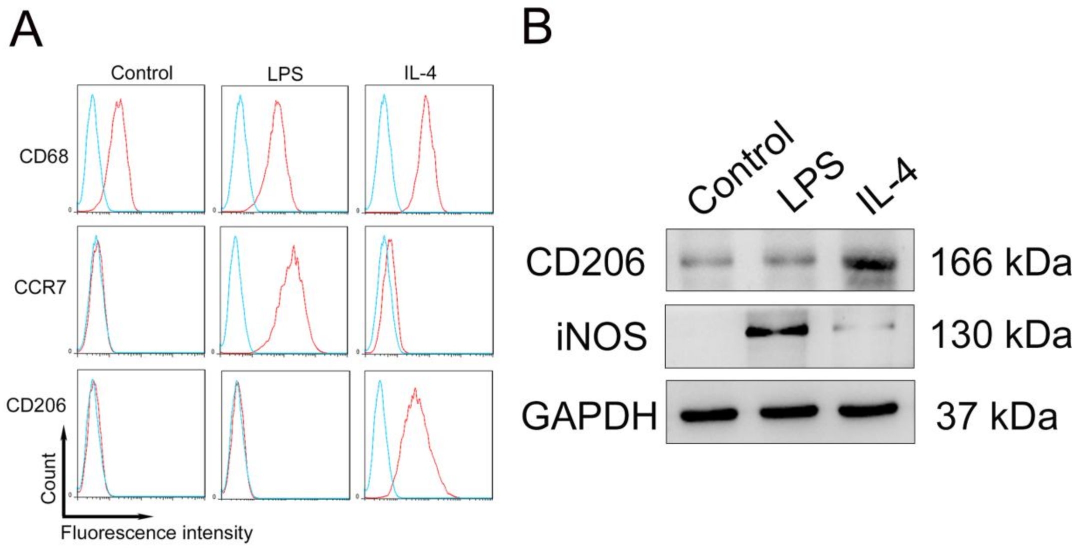

Supplementary Fig.3 Cytokine-induced macrophage polarization. Mouse macrophages (RAW 264.7) were cultured with lipopolysaccharide (LPS) or IL-4 (10 ng/ml) for 24 h. (A) Validation of M1 and M2 polarization by the flow cytometry. The M0 macrophages were characterized by flow cytometry with CD68 staining. CCR7 and CD206 are specific surface markers of M1 and M2 macrophages, respectively. The isotype control (blue) and indicated surface marker (red) was merged, 1×10^4 cells were counted per experiment. (B) Expression of CD206 and iNOS on macrophages was measured by western blotting.

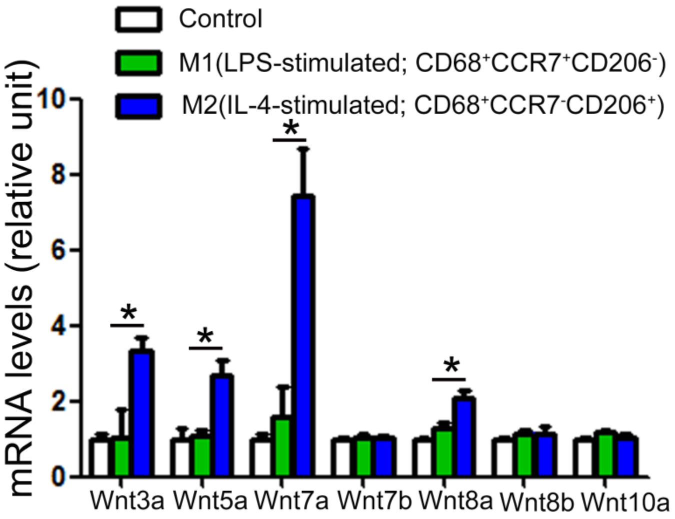

Supplementary Fig.4 M2 macrophages express significant levels of Wnt7a. RAW 264.7 cells were treated with LPS (10 ng/ml) or IL-4 (10 ng/ml) for 24 h to induce M1 and M2 macrophage differentiation, respectively. Expression of indicated cytokines in differentiated macrophage subtypes were determined by q-PCR. Results are expressed as means ± SD (n = 5; *$p < 0.05$ vs. M1 macrophage).

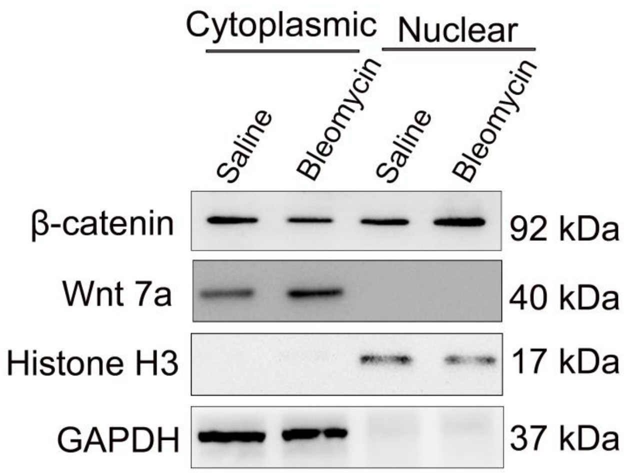

Supplementary Fig.5 Bleomycin treatment upregulates Wnt7a, and activates Wnt/β-catenin signaling in mouse lung tissues. The expression of β-catenin and Wnt7a in nuclear and cytoplasmic extracts was analyzed by western blotting. Histone H3 and GAPDH were used as loading controls for nuclear and cytoplasmic proteins, respectively.

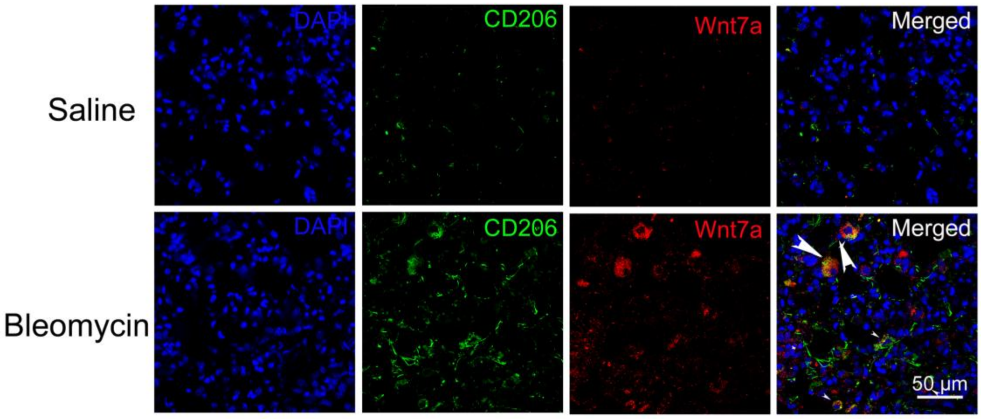

Supplementary Fig.6 M2 macrophages express Wnt7a in fibrotic lungs of bleomycin-treated mice. Colocalization of Alexa Fluor 488-labeled CD206 (green) and Alexa Fluor 594-labeled Wnt7a (red) was examined by a confocal fluorescence microscope. Arrow indicates individual cells positive for both CD206 (green) and Wnt7a (red).

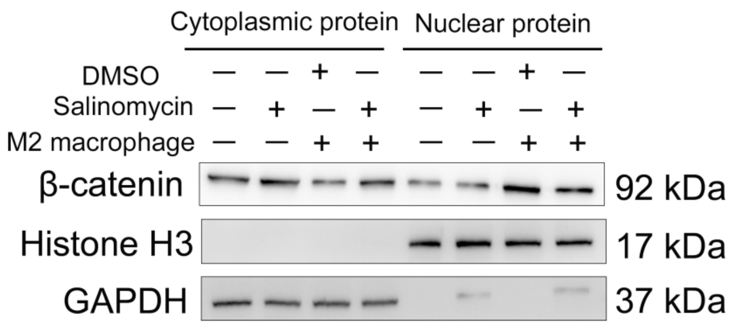

Supplementary Fig.7 Effects of salinomycin on the nuclear translocation of β-catenin induced by M2 macrophages in LR-MSCs. Cytoplasmic and nuclear extracts were prepared and analyzed by western blotting using β-catenin antibodies. Histone H3 and β-actin were used as loading controls for nuclear and cytoplasmic proteins, respectively.
